# Accurate reconstruction of bacterial pan- and core genomes with PEPPAN

**DOI:** 10.1101/gr.260828.120

**Published:** 2020-11

**Authors:** Zhemin Zhou, Jane Charlesworth, Mark Achtman

**Affiliations:** Warwick Medical School, University of Warwick, Coventry CV4 7AL, United Kingdom

## Abstract

Bacterial genomes can contain traces of a complex evolutionary history, including extensive homologous recombination, gene loss, gene duplications, and horizontal gene transfer. To reconstruct the phylogenetic and population history of a set of multiple bacteria, it is necessary to examine their pangenome, the composite of all the genes in the set. Here we introduce PEPPAN, a novel pipeline that can reliably construct pangenomes from thousands of genetically diverse bacterial genomes that represent the diversity of an entire genus. PEPPAN outperforms existing pangenome methods by providing consistent gene and pseudogene annotations extended by similarity-based gene predictions, and identifying and excluding paralogs by combining tree- and synteny-based approaches. The PEPPAN package additionally includes PEPPAN_parser, which implements additional downstream analyses, including the calculation of trees based on accessory gene content or allelic differences between core genes. To test the accuracy of PEPPAN, we implemented SimPan, a novel pipeline for simulating the evolution of bacterial pangenomes. We compared the accuracy and speed of PEPPAN with four state-of-the-art pangenome pipelines using both empirical and simulated data sets. PEPPAN was more accurate and more specific than any of the other pipelines and was almost as fast as any of them. As a case study, we used PEPPAN to construct a pangenome of approximately 40,000 genes from 3052 representative genomes spanning at least 80 species of *Streptococcus*. The resulting gene and allelic trees provide an unprecedented overview of the genomic diversity of the entire *Streptococcus* genus.

Soon after the first bacterial genome was sequenced (Fleischmann et al. 1995), it became clear that the genomic contents varied between individual strains within a prokaryotic species. Variable genomic content is caused by the gain or loss of singleton ORFan genes (Daubin and Ochman 2004), genomic islands, selfish DNA (plasmids, bacteriophages, integrative conjugative elements), and/or widespread horizontal gene transfer (HGT) (Abby et al. 2012; Szöllösi et al. 2012; Croucher et al. 2014). Thus, the designation “pangenome” was introduced to refer to the entire gene contents of a bacterial species or set of strains (Tettelin et al. 2005). Bacterial pangenomes can be divided into the core genome, which consists of the subset of genes that are present in all genomes, and the accessory genome, which consists of genes that are variably present among individual genomes. The core genome often contains phylogenetic signals reflecting the vertical accumulation of mutations and can be used for assignments of bacterial strains to populations.

An early genomic comparison of eight strains of *Streptococcus agalactiae* indicated that for some bacterial species, the total size of the pangenome may increase indefinitely with the number of genomes sequenced, a concept dubbed an “open” pangenome (Tettelin et al. 2005). The validity of this concept remains questionable because, until recently, few pangenome analyses have included more than 100 genomes (Vernikos et al. 2015), in part because only a limited number of bacterial genomes had been sequenced. Furthermore, initial pangenome construction algorithms (OrthoMCL [Li et al. 2003]; Panseq [Laing et al. 2010]; PGAP [Zhao et al. 2012]) were incapable of handling larger numbers of genomes as they rely on an initial all-against-all sequence comparison, which scales computationally with the squared number of gene sequences.

The insufficiency of data no longer exists, as bacterial genome assemblies now number in the 100,000s for some genera (Sanaa et al. 2019; Zhou et al. 2020). However, such large numbers of genomes exacerbate the scalability problem. Fortunately, at least three recent pipelines (Roary [Page et al. 2015]; panX [Ding et al. 2018]; PIRATE [Bayliss et al. 2019]) exist for constructing pangenomes from large and representative data sets (Alikhan et al. 2018; Ding et al. 2018).

However, pangenome construction from large data sets is still hampered by two problems. First, both genome annotations in public repositories and those from automatic annotation pipelines such as PROKKA (Seemann 2014) are incomplete and inconsistent (Denton et al. 2014; Wozniak et al. 2014; Salzberg 2019). These inconsistencies are propagated into genomic studies and can confound further analyses. Early pangenome analyses (Tettelin et al. 2005; Hogg et al. 2007) addressed these problems by running TBLASTN gene-against-genome comparisons, but such inconsistencies between genome annotations are not addressed by the latest generation of pangenome pipelines, which do not include a reannotation step. Namely, genes that have been fragmented by assembly errors or pseudogenization may still be relevant to cell function (Goodhead and Darby 2015) and should therefore be included in pangenomes. The identification of such gene fragments requires comparisons against intact analogs (Lerat and Ochman 2005), but automatic annotation pipelines instead annotate them as multiple intact genes, reducing the size of the estimated core genome and overestimating the overall size of the pangenome.

The second problem in computing a pangenome is that of differentiating orthologous genes, which have evolved by vertical descent, from paralogous genes derived from gene duplications or HGT events. Paralogous genes can become fixed in populations, but many are gained or lost multiple times. This generates complex patterns of presence/absence along the phylogeny. Therefore, including paralogous genes in a phylogenetic analysis may lead to inaccurate interpretations. State-of-the-art pangenome pipelines implement either graph- or tree-based algorithms for the identification of paralogous genes (Altenhoff et al. 2019). However, tree-based algorithms (used by panX) that reconcile gene trees with a species tree do not scale well to large data sets. Graph-based algorithms (used by Roary and PIRATE) run faster because they ignore phylogenetic relationships between genomes but perform poorly on benchmark data sets (Ding et al. 2018).

Here we present PEPPAN, a novel pipeline for calculating pangenomes that specifically deals with the problems described above. We describe the algorithms implemented within PEPPAN and show that it outperforms other pangenome methods on both empirical and simulated data sets. As a demonstration of PEPPAN's capabilities, we present a pangenome calculated from 3052 representatives of *Streptococcus*, a highly diverse genus.

## Results

### A brief overview of PEPPAN

**Figure 1.**
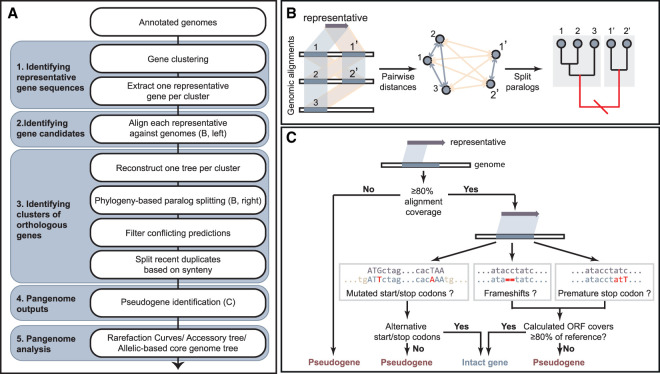
A brief overview of the workflow for PEPPAN. (*A*) Flow chart indicating the five cascading groups of operations (blue-gray) from *top* to *bottom*. (*B*) Cartoon of similarity-based prediction of gene candidates (*left*) and phylogeny-based paralog splitting (*middle* and *right*). The tree was split at the red branch (*right*) to separate gene candidates into two subclusters. The gene pairs in the same subcluster had low paralogous scores (blue-gray quadrilaterals [*left*] and arrows [*middle*]), whereas gene pairs between the subclusters had high paralogous scores (yellow). (*C*) Flow chart of the pseudogene identification. The detailed workflow of the algorithm implemented in PEPPAN can be found in Supplemental Fig. S1 and Supplemental Text 1.

PEPPAN's workflow consists of the following five successive groups of operations (Fig. 1A; Supplemental Fig. S1) with additional details in Supplemental Text 1.

1. Identifying representative gene sequences. The inputs for PEPPAN consist of GFF3 formatted genome assemblies (https://www.ensembl.org/info/website/upload/gff3.html). PEPPAN also accepts inputs of additional nucleotide sequences, which are used to refine gene predictions. To reduce the number of genes used in downstream analyses, PEPPAN iteratively clusters genes using Linclust (Steinegger and Söding 2017), resulting in a single representative gene sequence per 90% nucleotide homology cluster.
2. Identifying gene candidates. Each representative gene is aligned to all genomes using both BLASTN (Altschul et al. 1990), which accurately locates short inserts and deletions (indels), and DIAMOND (Buchfink et al. 2015), which generates amino acid alignments and has greater sensitivity with divergent sequences than BLASTN. Alignments are rescored, and all sequences with homology ≥50% across ≥50% of the representative sequence (Supplemental Text 1.2) are clustered in a neighbor-joining tree using RapidNJ (Simonsen et al. 2011).
3. Identifying clusters of orthologous genes. PEPPAN identifies putative orthologs by calculating a paralogous score for each branch in a gene cluster tree (see Supplemental Text 1.3.2) based on ratio of the pairwise genetic distances of candidate genes within each cluster to the average genetic distances of their host genomes (Fig. 1B). Using average genetic distances avoids potential errors that can be introduced by using a “species” tree to reconcile individual gene cluster trees (Altenhoff et al. 2019). Branches with a paralogous score of greater than one are iteratively pruned until none remain. The remaining monophyletic subtrees are treated as putative orthologs.
  The genomic locations of multiple putative orthologs may overlap in some genomes owing to either inconsistent genome annotations or a failure to cluster divergent orthologous sequences in the first stage. These conflicts are resolved by retaining the ortholog with the greatest information score (see Supplemental Text 1.3.3) and eliminating all other gene candidates for that region.
  The remaining gene candidates from each genome are ordered according to their genomic coordinates, and the final set of orthologous genes is identified based on synteny (see Supplemental Text 1.3.4).
4. Pangenome outputs. Each gene candidate in each genome is categorized as either an intact coding sequence (CDS) or a pseudogene, depending on the size of the aligned reading frame relative to its representative gene (Fig. 1C). It is also possible to predict pseudogenes that are disrupted in all genomes by importing their intact analog into PEPPAN as an external representative gene. Finally, the evaluations of all genes, as well as their genomic coordinates and orthologous group, are output in GFF3 format, and the extents of the regions that match to their representative genes are saved in FASTA format.
5. Pangenome analysis. A separate tool, PEPPAN_parser, generates analyses of the estimated pangenome based on the GFF3 outputs from PEPPAN (details can be found at https://github.com/zheminzhou/PEPPAN/blob/master/docs/source/usage/outputs.rst). Similar to Roary (Page et al. 2015) and PIRATE (Bayliss et al. 2019), these include rarefaction curves, gene presence matrices, and gene presence trees. In addition, PEPPAN_parser can also calculate a core genome tree based on allelic differences of genes that are conserved in most genomes. These core genome trees can scale to 10,000s of genomes and provide the basis for all core genome MLST schemes in EnteroBase (Supplemental Text 3; Zhou et al. 2020).

### Comparisons of PEPPAN with state-of-the-art pangenome pipelines

We assessed the absolute performance of PEPPAN and compared it with other, recently described pipelines for pangenome construction (Roary [Page et al. 2015]; panX [Ding et al. 2018]; PIRATE [Bayliss et al. 2019]), as well as with a classical, small-scale pipeline (OrthoMCL [Li et al. 2003]).

It is important to examine multiple aspects of genomic diversity for these comparisons because the evolutionary history of bacterial pangenomes can be highly complex. However, we are not aware of any prepackaged simulation tools that can encompass the entire diversity of bacterial genomic changes, including gene duplications and HGTs (leading to paralogs), homologous recombination, and large-scale gene insertions and deletions. We therefore performed our first benchmarks by comparing a pangenome calculated from 15 manually curated *Salmonella enterica* genome annotations (Nuccio and Bäumler 2014) with pangenomes based on automated annotations of the same genome assemblies. Subsequently, we designed a new simulation tool, SimPan, which uses SimBac (Brown et al. 2016), to simulate the dynamics of pangenome evolution via recombination, HGT, and gene gain and loss, as well as the creation of paralogs (Supplemental Text 2).

### Benchmarking pangenome pipelines on 15 curated genomes

Nuccio and Bäumler reannotated 15 complete genomes of *S. enterica* (Nuccio and Bäumler 2014). They removed existing annotations for unreliable short genes, performed new BLASTN and TBLASTN alignments to identify previously not annotated genes, corrected the start positions of falsely annotated genes, and predicted the existence of pseudogenes based on alignments with orthologous intact CDSs. The result of these efforts is a unique set of consistently annotated genomes from a single species, which we equated with the “ground truth” with which to compare the results from the pangenome pipelines.

First, we compared the manual reannotation with three sets of gene annotations for each of the 15 *S*. *enterica* genomes: (1) the original annotation that had been submitted to NCBI GenBank (https://www.ncbi.nlm.nih.gov/genbank/) (“Submitter”), (2) an automated reannotation from RefSeq (Haft et al. 2018) that was generated with PGAP (Tatusova et al. 2016), and (3) a novel annotation using PROKKA (Seemann 2014), another popular bacterial annotation pipeline. Genes that had been eliminated by Nuccio and Bäumler as being “unreliable” were removed from all three annotations for consistency. We then examined the degree of concordance between the pangenome published by Nuccio and Bäumler with the pangenomes calculated by each of the pipelines. Concordance was estimated by calculating the adjusted Rand index (ARI) (Rand 1971), which is a measure of similarity between clustering results. For Roary or PIRATE, we only report results from the run with the greatest ARI among three parallel runs with varying minimum identity (50%, 80%, or 95%), because the optimal value of this parameter differs for various levels of diversity (Ding et al. 2018; our own observations).

**Figure 2.**
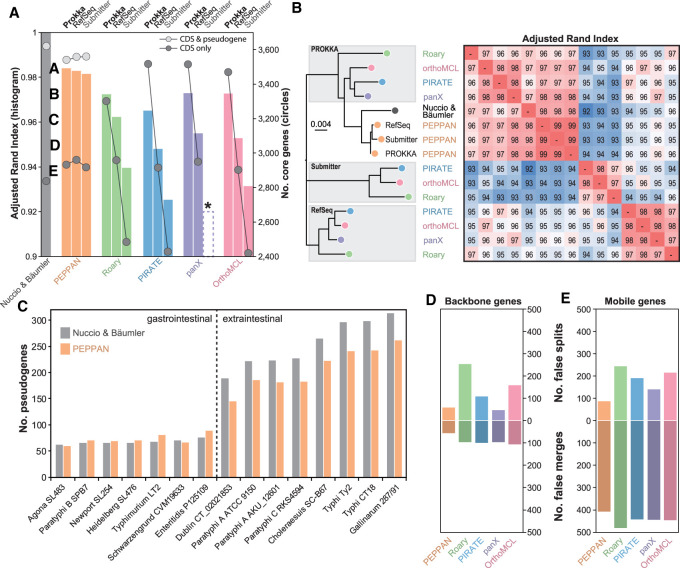
Comparison of pangenome predictions for 15 *Salmonella* genomes with a manually curated pangenome (Nuccio and Bäumler 2014). (*A*) The adjusted Rand index versus the manual curation (ARI; histogram) and the sizes of core genomes (circles) in each of the pangenomes after annotation by PROKKA (Seemann 2014), after reannotation in RefSeq with PGAP (Tatusova et al. 2016), and as originally submitted to NCBI (Submitter). An asterisk indicates that panX failed to run on the “Submitter” annotations. (*B*) A neighbor-joining tree (*left*) of the pairwise ARI scores (heatmap at the *right*) between the predicted pangenomes and the curated pangenome. The annotation source is indicated within gray shadows at the *left* except for PEPPAN, where it is listed at the tips. Colors are as in *A*. (*C*) Histogram of the numbers of pseudogenes (*y*-axis) in each of the genomes (*x*-axis) in the curated pangenome (gray) and pangenome predicted by PEPPAN (orange). A dashed line separates the two *Salmonella* pathovar groups described by Nuccio and Bäumler. (*D*,*E*) Histograms of the average numbers of false splits (*top*) and merges (*bottom*) of ortholog groups by the individual pipelines (*x*-axis) in backbone (*D*) or mobile (*E*) genes.

All pipelines successfully calculated a pangenome from each of the four annotations, except that “Submitter” annotations never ran to completion with panX. The PEPPAN pangenomes consistently yielded ARIs of ∼0.98 relative to the manual pangenome (Fig. 2A, histograms). This is not surprising because PEPPAN recalculates gene annotations in a fashion that resembles that of the manual curation. All the other pipelines yielded lower ARI values that varied between the annotation methods. The PROKKA annotations yielded ARIs of 0.97 with Roary, panX, and OrthoMCL and 0.96 with PIRATE. The ARIs were 0.95–0.96 for the PGAP annotations from RefSeq and 0.93–0.94 for the “Submitter” annotations. We also performed hierarchical clustering using the neighbor-joining algorithm on pairwise comparisons of the ARI scores across all 14 pangenomes (Fig. 2B). The three pangenomes predicted by PEPPAN formed a tight cluster with high pairwise ARI (0.99), which clustered tightly with the curated pangenome (ARI = 0.98). In contrast, pangenomes generated by the other pipelines clustered according to annotation source rather than pipeline methodology. For each of the three annotation sources, the pangenome predicted by Roary was the most distinct, whereas pangenomes predicted by OrthoMCL, panX, and PIRATE clustered more tightly. These results may reflect the fact that Roary differs from the other pipelines by performing an additional splitting of paralogs on the basis of synteny.

#### Pseudogene prediction

The core genome defined by Nuccio and Bäumler contained 2838 CDSs that were intact in all 15 genomes and 783 others that were disrupted in at least one genome. PEPPAN predicted marginally more intact CDSs, and slightly fewer pseudogenes, from all three annotations than were present in the manual annotations (Fig. 2A, circles). The number of pseudogenes for each genome was also very similar between the manual curations and PEPPAN's automated predictions. We note that PEPPAN consistently predicts fewer pseudogenes for extraintestinal strains than those for those linked to gastrointestinal disease (Fig. 2C), This is an interesting observation, as accumulation of pseudogenes has been linked to host specialization in *Salmonella* (Parkhill et al. 2001; Holt et al. 2008; Nuccio and Bäumler 2014; Zhou et al. 2014, 2018b).

Roary, OrthoMCL, and panX do not predict any disrupted genes. PIRATE reports “gene diffusion,” a measure of the frequency with which CDSs that are intact in some genomes are split into two or more fragments in others. However, it did not detect any gene diffusion in the RefSeq and GenBank annotations, as well as only one instance with the PROKKA annotations. PIRATE also failed to predict fragmented genes. Similar to the ARI comparisons described above, the total numbers of predicted core CDSs varied according to annotation source for all pipelines other than PEPPAN. The four pipelines reported 3301–3515 core CDSs from PROKKA annotations (Fig. 2A, right). These numbers are similar to the total number of intact core CDSs plus pseudogenes within the curated pangenome, indicating that PROKKA predicted many pseudogenes as intact CDSs. Roary, PIRATE, and OrthoMCL only detected 2418–2484 core genes in the originally submitted genomes, suggesting inconsistencies between individual genome annotations. In contrast, all four pipelines predicted 2901–2957 core CDSs from the RefSeq annotations, and these numbers were similar to the numbers of intact core CDSs in the curated pangenome (2838) or as predicted by PEPPAN (2918–2961).

#### Inaccurate prediction of orthologs

Inconsistent ortholog calls relative to the manually curated pangenome (Nuccio and Bäumler 2014) also contributed to variation in the numbers of core CDS predicted by the different pipelines. We designated as “false splits” those cases in which a single ortholog cluster in the curated pangenome was split into multiple ortholog clusters by a pipeline. Similarly, “false merges” occurred when multiple orthologous clusters in the curated pangenome were assigned to a single orthologous cluster. We identified 4695 “backbone genes” in the curated pangenome that were present in the most recent common ancestor (MRCA) and 3364 “mobile” genes, which were associated in one or more genomes with mobile genetic elements and which were absent from the MRCA. For backbone genes, PEPPAN made the fewest false splits and false merges of all five pipelines, followed by panX (Fig. 2D). False merges were made four times as often by all pipelines for mobile genes than for backbone genes, and false splits were up to two times as frequent (Fig. 2, cf. E and D). Roary generated the highest number of false calls, whereas PEPPAN generated the lowest.

### Simulating pangenome data sets

The analyses above indicate that the backbone and mobile genes might differ in their rates of gain and loss during evolution. To test the abilities of pangenome pipelines to handle varying rates of gene gain and loss, we created SimPan (https://github.com/zheminzhou/SimPan) to simulate the evolution of real bacterial pangenomes (Supplemental Fig. S2; Supplemental Table S1; for details, see Supplemental Text 2). In brief, SimPan uses SimBac (Brown et al. 2016) to generate a clonal genomic phylogeny. This clonal phylogeny is subjected to random homologous recombination, resulting in different “local trees” that reflect the individual ancestries of backbone and mobile genes. Random indel events leading to loss or gain of blocks of genes are simulated along the branches of these local trees until the average number of genes per genome and in the core genome attain user-specified parameters “‐‐aveSize” and “‐‐nCore” (Supplemental Table S1). This results in a presence/absence matrix of all backbone and mobile genes. Finally, sequences of both genes and intergenic regions are subjected to short indels, converted into genes with INDELible (Fletcher and Yang 2009), and concatenated into whole genomes.

**Figure 3.**
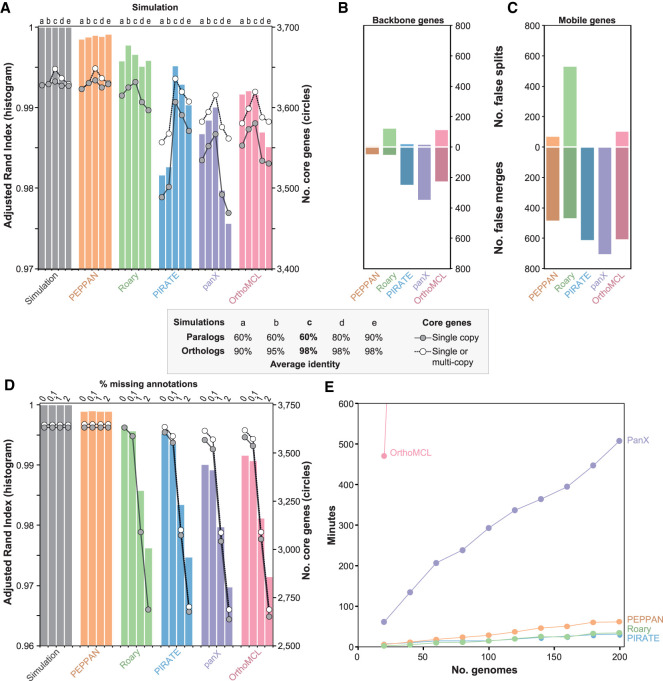
Comparison of the pangenome pipelines with simulated data generated by SimPan. (*A*) The adjusted Rand index (ARI; histogram) and the sizes of core genomes (circles) in the pangenomes produced by SimPan simulations a, b, c, d, e (*inset*). (*Left*) Pangenome produced by the simulations. Other histograms, pangenomes calculated by five pipelines. (*B*) Numbers of failed splits (*top*) and false merges (*bottom*) of ortholog groups by five pipelines with backbone genes. (*C*) Numbers of failed splits (*top*) and false merges (*bottom*) of ortholog groups by five pipelines with mobile genes. (*D*) The adjusted Rand index (ARI; histogram) and the sizes of core genomes (circles) in the pangenomes produced by SimPan simulation c after random deletions of 0%, 0.1%, 1%, and 2% of the gene annotations. Other details as in *A*. (*E*) Runtime for each pipeline (*y*-axis) versus number of genomes in simulated data sets (*x*-axis). Runs that exceed 600 min are not shown.

We simulated five genomic data sets each containing 15 genomes, using parameters derived from the curated *S. enterica* pangenome, with each genome containing a mean of 3621 core genes and 879 accessory genes (simulations a–e). We arbitrarily assigned 5% of the backbone genes and 40% of the mobile genes to paralogous clusters and varied their mean percentage sequence identities between each set of simulations (Fig. 3, inset). Simulation c represents the simplest pangenome construction scenario, with high sequence identity (98%) between genes in an ortholog cluster and low sequence identity (60%) between genes in a paralog cluster. Simulations a and b have decreasing levels of identity between orthologs to simulate more diverse species, whereas simulations d and e have increasing levels of identity between paralogs in order to simulate recent gene duplications.

### Pipeline performance on simulated genomes

Pangenomes calculated from each simulated data set by PEPPAN, Roary, PIRATE, panX, and OrthoMCL were compared with the original pangenomes produced by SimPan (Fig. 3A). Once again, PEPPAN pangenomes were highly concordant with the known truth (ARI ≥ 0.998 for all comparisons). Roary performed comparably to PEPPAN on all simulated data sets (ARI ≥ 0.995). PIRATE performed almost as well on simulations c to e but yielded ARI scores below 0.99 when run on simulations of more diverse genomes (simulations a and b). In contrast, panX and OrthoMCL yielded ARI scores ≥0.99 when run on simulations a and b but were less concordant (ARI < 0.99) when run on simulations containing more recent gene duplications (simulations d and e).

PEPPAN correctly predicted all core genes in simulations b, c, and e, and only missed two to three core genes in the two remaining data sets (Fig. 3A, circles). Roary correctly predicted all single-copy core genes for simulation c but failed to identify any multicopy core genes for any data set, likely owing to its aggressive synteny-based paralog identification step. PIRATE, panX, and OrthoMCL significantly underestimated the number of core genes when only single-copy core genes were counted, suggesting a high frequency of false splitting of paralog clusters. Indeed, the frequency of false merges was particularly high for backbone genes with these three pipelines, and the frequency of false splits was high with Roary and OrthoMCL (Fig. 3B). All pipelines made multiple false merges of mobile genes, possibly because of their predominance among paralog clusters, and Roary also made large numbers of false splits (Fig. 3C). Overall, PEPPAN made the fewest false calls for both backbone and mobile genes, which explains its higher ARI scores.

#### The effects of missing gene annotations on the pangenome

As shown above, inconsistent or inaccurate gene annotations are problematic for calculating reliable pangenomes. We simulated this effect by randomly deleting 0.1%, 1%, or 2% of the gene annotations from simulation c (Fig. 3D). Because PEPPAN reassigns individual genes to ortholog clusters, it was unaffected by these manipulations. However, the missing annotations yielded drastically reduced ARI scores (Fig. 3D. histograms) and core genome sizes (Fig. 3D, circles) for the other pipelines, and ARI scores became progressively worse with the proportion of missing annotations.

#### Computation time

We generated 10 additional simulations of 20–200 genomes with the same parameters as simulation c and measured the running wall times to calculate a pangenome for all five pipelines using four processors on a server with 1 TB of memory and 40-CPU cores (Fig. 3E). OrthoMCL was the slowest and needed >24 h for 60 or more genomes. panX was at least eightfold slower than the other three pipelines and needed 500 min for 200 genomes, despite using a divide-and-conquer algorithm on data sets with more than 50 genomes. Both Roary and PIRATE scaled very well, and each completed the calculations on 200 genomes within 30 min. PEPPAN is about twice as slow as either Roary or PIRATE and needed 63 min for 200 genomes. The good scalability of these pipelines is likely related to the preclustering step, which reduces the number of genes used in downstream comparisons. However, this preclustering step becomes less efficient with increasing genetic diversity: In an independent simulation of 200 genomes with only 90% sequence identity, the runtime for all three pipelines increased by at least twofold relative to simulation c (PEPPAN: 144 min; Roary: 132; PIRATE: 60).

### A pangenome for the genus *Streptococcus*

PEPPAN can construct a pangenome from thousands of genomes with high genetic diversity, and earlier versions of this pipeline were used to generate cgMLST schemes for the genera represented in EnteroBase (Alikhan et al. 2018; Frentrup et al. 2020; Zhou et al. 2020), as well as for ancient DNA analyses (Zhou et al. 2018b; Achtman and Zhou 2020). To show PEPPAN's capability on genetically diverse data sets, we chose the genus *Streptococcus*, which includes highly significant zoonotic and human pathogens (Gao et al. 2014).

**Figure 4.**
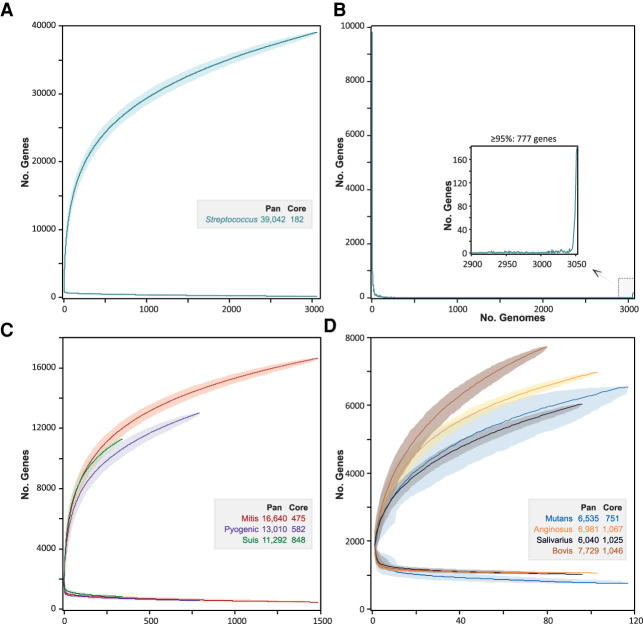
Rarefaction curves of pangenomic and core gene numbers in *Streptococcus* and its seven major taxonomic subgroups. (*A*) Rarefaction curves created with PEPPAN_parser for the accumulations of pan genes and core genes of 3052 *Streptococcus* representative genomes from 1000 random permutations. (*B*) The frequencies of pan genes (*y*-axis) by the numbers of genomes that carried that many genes (*x*-axis). The *inset* shows the relaxed core genes present in ≥95% of the genomes. (*C*) Rarefaction curves of genomes in the Mitis, Pyogenic, and Suis groups. (*D*) Rarefaction curves of genomes in the Mutans, Anginosus, Salivarius, and Bovis groups. The dark lines in *A*, *C*, and *D* indicate median values and the shadows indicate 95% confidence intervals.

We generated a data set of 3052 high-quality genomes (Supplemental Table S2A) representing the entire taxonomic diversity of *Streptococcus* (see Methods). PEPPAN took 5 d to construct a pangenome from this data set. The resulting pangenome contained 39,042 genes, twice as many as a previous pangenome based on 138 *Streptococcus* genomes (Gao et al. 2014). In agreement with the earlier conclusions by Gao et al., the rarefaction curve showed no sign of plateauing, and the pangenome continued to expand with each new genome added (Fig. 4A). Gao et al. estimated that the pangenome would expand by 62 genes for each new genome, whereas we estimate a lower rate of 39 genes per new genome for a randomly sampled set of 138 genomes. However, the growth rate dropped with the increased number of genomes, and we estimate that the future expansion rate of the pangenome is only 4.4 new genes for every newly added genome.

In contrast to earlier studies (Gao et al. 2014), which defined a strict core genome of 278 orthologs, we found only 182 genes that were shared across all *Streptococcus* genomes (Fig. 4B, inset). Each of these was disrupted in at least one of the 14,115 *Streptococcus* genomes in RefSeq. This is a common problem for core genome analyses, especially because the multiple contigs within draft genomes can result in the absence of multiple genes from genome assemblies. Core genome schemes used for cgMLST are therefore usually based on a relaxed core, consisting of single-copy genes present in the large majority of representative isolates (Moura et al. 2017; Alikhan et al. 2018; Zhou et al. 2020). Our analyses identified 754 genes that were present in at least 2900 (95%) of the representative streptococcal genomes (Supplemental Table S3, Fig. 4B). However, most of the 754 genes were present in multiple copies in some genomes, leaving a final relaxed core of 292 single-copy genes that are suitable for identifying core genomic relationships and evolutionary history (Table 1; Supplemental Table S3).

**Table 1.**
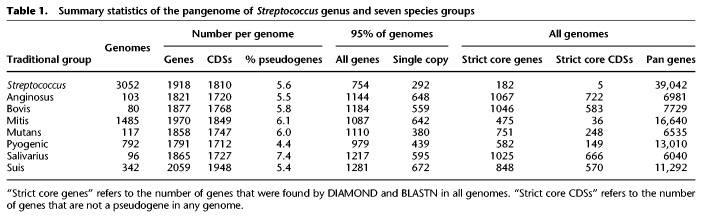
Summary statistics of the pangenome of *Streptococcus* genus and seven species groups

### Taxonomic clusters within *Streptococcus*

*Streptococcus* taxonomy is a highly dynamic area of research (Kikuchi et al. 1995; Jensen et al. 2016; Dekker and Lau 2016; Velsko et al. 2018, 2019; Kilian and Tettelin 2019; Zhou et al. 2020). Many *Streptococcus* species are currently defined exclusively by phenotypic markers, and multiple taxonomic assignments in RefSeq are incorrect (Beaz-Hidalgo et al. 2015; Gomila et al. 2015; Kilian and Tettelin 2019). We therefore initially ignored taxonomic designations and used the normal cut-off of ANI ≥95% as a proxy for species designations (Konstantinidis et al. 2017; Jain et al. 2018). Single-linkage agglomerative clustering of pairwise ANI values calculated from the 3052 representative genomes revealed 223 clusters (Supplemental Table S2). For the 29 clusters containing 10 or more genomes, we also identified a dominant species designation from NCBI metadata, as shown in Supplemental Table S4. Information on each cluster's pangenome can be found in Supplemental Text 4 and Supplemental Table S5.

**Figure 5.**
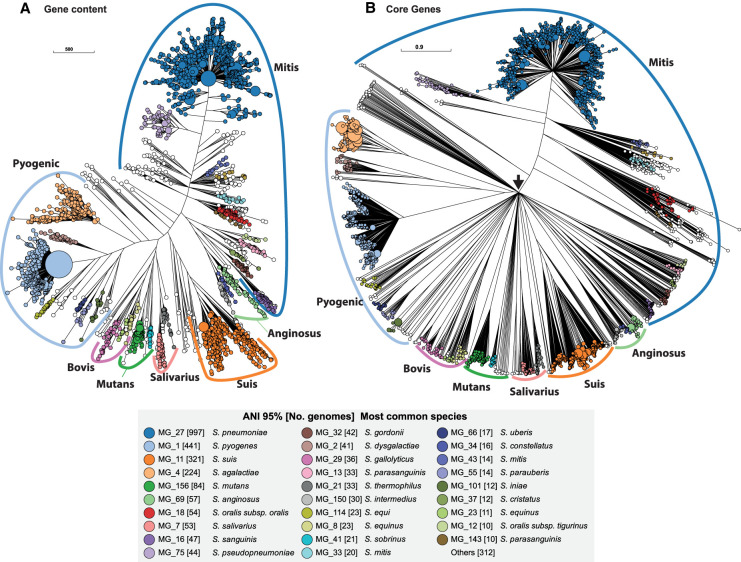
Phylogenies of 3052 *Streptococcus* genomes based on accessory gene content (*A*) and allelic variation in relaxed core genes (*B*). (*A*) A FastTree (Price et al. 2010) phylogeny based on binary information of the presence and absence of accessory genes. (*B*) A RapidNJ (Simonsen et al. 2011) phylogeny based on numbers of identical sequences (alleles) of 292 single copy, relaxed, core genes that are present in ≥95% of *Streptococcus* genomes. These trees are represented in GrapeTree (Zhou et al. 2018a). The sizes of the circles in *A* and *B* are proportional to the numbers of genomes they encompass and are color-coded by 29 common ANI95% clusters as shown in the *inset*. Many *Streptococcus* species have been assigned to one of six traditional taxonomic groups whose names are shown *outside* colored arcs. These trees define from the Suis group which contains *Streptococcus suis*. A black arrow in *B* shows the root of the tree, where multiple branches radiate directly outward owing to lack of resolution of cgMLST for such distant taxa. All ANI95% cluster information can be found in Supplemental Table S4. Interactive versions of the trees can be found at (*A*) https://achtman-lab.github.io/GrapeTree/MSTree_holder.html?tree=https://raw.githubusercontent.com/zheminzhou/PEPPA_data/master/Strep.content.json and (*B*) https://achtman-lab.github.io/GrapeTree/MSTree_holder.html?tree=https://raw.githubusercontent.com/zheminzhou/PEPPAN_data/master/Strep.CGAV.json.

We used PEPPAN_parser to generate two trees of the 3052 representative genomes based on the presence or absence profiles of 39,042 pan genes (Fig. 5A) and on the allelic variation profiles of 292 relaxed core genes (Fig. 5B). The topology of the first tree reflects similarities in pangenome content, and the topology of the second tree reflects sequence similarities within core genes. The details of these two topologies differed somewhat. In particular, the core gene tree contained an unresolved, star-like radiation that we attribute to distinct sequences in all of the core genes from highly diverse species. However, despite these differences in deep branching topology, both trees showed comparable tight clustering of genomes corresponding to each of the 29 common taxonomic groupings. This tight clustering indicates that the topologies of both trees are congruent at the ANI95% level. Both trees also support published taxonomic assignments of subspecies. For example, MG_29 corresponds to *Streptococcus gallolyticus* and includes its three subspecies *gallolyticus*, *macedonicus*, and *pasteurianus* (Dekker and Lau 2016). Similarly, MG_2 corresponds to *Streptococcus dysgalactiae* and includes its two subspecies *dysgalactiae* and *equisimilis* (Jensen and Kilian 2012).

Both *Streptococcus* trees also clustered high order branches according to the traditional taxonomical group names Mitis, Anginosus, Salivarius, Mutans, Bovis, and Pyogenic (Gao et al. 2014). They clustered *Streptococcus suis* together in a seventh phylogenetic branch, which we designate as Suis, and also clustered *Streptococcus acidominimus*, *Streptococcus minor*, *Streptococcus hyovaginalis*, *Streptococcus ovis*, and multiple other taxa into a novel, unnamed neighboring branch. By using PEPPAN_parser, we calculated a pangenome for each of the seven named taxonomical groups. Similar to the *Streptococcus* pangenome, each group pangenome is open (Fig. 4C,D) and grows at the rate of 3.5–30.1 new genes for each new representative genome. Unlike the entire genus, these seven named taxonomic groups possess a sizable strict core genome, consisting of 475–1067 core genes (Fig. 4C,D; Table 1). After excluding multicopy genes, the sizes of the group-specific, 95% relaxed core genomes ranged from 380 (Mutans) to 672 (Suis) genes (Fig. 4C,D; Table 1).

In accord with prior observations (Kilian et al. 2008; Kilian and Tettelin 2019), numerous discrepancies differentiate the ANI95% groups and the taxonomic designations in RefSeq. Some discrepancies reflect inaccurate metadata, but others reflect true discrepancies between ANI95% clusters and taxonomic designations made by expert microbiologists. For example, *Streptococcus mitis* spans 44 distinct ANI95% clusters (Fig. 5; Supplemental Table S4). Similarly, *Streptococcus oralis* straddles multiple, distinct ANI95% clusters, as did each of the three *S. oralis* subspecies *oralis, tigurinus*, and *dentisani* defined by Jensen et al. (2016). Further investigations will be needed to elucidate how many biological species are truly present within the genus *Streptococcus.* We anticipate that the trees in Figure 5 might be useful for such analyses.

## Discussion

### Comparison of PEPPAN with other pangenome pipelines

Pangenome pipelines must be efficient in order to handle the computational demands of modern, large-scale comparative genomics. Roary (Page et al. 2015) and PIRATE (Bayliss et al. 2019) were the fastest of all the pipelines tested, likely reflecting their choice of time efficient approaches in every stage of their algorithms. However, this speed comes with trade-offs in terms of accuracy (Figs. 2, 3). The workflow implemented in PEPPAN requires many more calculations than other pipelines owing to its implementation of tree-based splitting of paralogs and similarity-based internal gene prediction but is only marginally slower because of the care that was taken to implement time efficient algorithms.

Roary, PIRATE, and PEPPAN all use a preclustering step to reduce the numbers of genes that are analyzed in subsequent, very time-consuming, all-against-all comparisons. PEPPAN accelerates this step by using Linclust (Steinegger and Söding 2018). Linclust scales linearly with the number of genes and is faster than CD-HIT, the clustering package used by Roary and PIRATE.

Roary and Pirate both use MCL, a graph-based clustering approach (Enright et al. 2002) to split paralogous clusters. MCL identifies a strict optimal threshold that separates orthologous genes from paralogous genes, and scales well with the numbers of genes. This approach is accurate for closely related genomes but is error-prone when data sets contain both closely related and distantly related genomes, because a single optimal clustering threshold does not exist for both extremes. PIRATE thus failed to split many paralogous clusters from real (Figs. 2D,E) and simulated (Fig. 3B,C) genomes, especially for more diverse data sets (Fig. 3A). Roary implements an additional synteny-based approach to identify and split unresolved paralog clusters, but this approach also failed to correctly split orthologs into multiple clusters (Figs. 2D,E, 3B,C). In contrast, PEPPAN identifies an optimal threshold for each gene and uses that threshold to split paralogous branches in the gene trees. This allows accurate estimates of pangenomes even in data sets of highly divergent genomes.

panX uses a “divide and conquer” strategy for the gene comparisons, which is computationally demanding. In addition, panX constructs a gene tree for every potential gene cluster, which, similar to other tree-based approaches, involves the alignment of gene sequences using MAFFT (Katoh and Standley 2013) followed by a tree construction using FastTree (Price et al. 2010). As a result, panX is substantially slower than PEPPAN, PIRATE, or Roary (Fig. 3E). However, panX was not substantially more accurate than those programs (Figs. 2A, 3A), which might be attributed to its use of raw pairwise genetic distances of genes for paralog splitting. In contrast, inspired by the methods used by large-scale genomics studies (Chewapreecha et al. 2014; Banaszkiewicz et al. 2019), PEPPAN uses a reference-based approach to generate an alignment for each gene group, which is then used to reconstruct a neighbor-joining gene tree using RapidNJ. These methods are less accurate but much faster than those in panX and scale to thousands of sequences. As a result, although the run time of PEPPAN was approximately twice as long as the run time of Roary or PIRATE, it still scaled linearly with the number of genomes (Fig. 3E).

### Effects of internal annotations by PEPPAN

Our benchmarking analyses on real and simulated genomes revealed the strong impact of inconsistent annotations on the pangenome predictions (Fig. 2A). Indeed, differences in annotation influenced the quality of the pangenome more than pipeline algorithms (Fig. 2B) and decreased the number of core genes by up to one-third for some pipelines (Fig. 2A). PEPPAN avoids this problem by implementing a similarity-based gene prediction step. Accordingly, pangenomes predicted by PEPPAN varied only slightly with different annotations (Fig. 2A,B). Draft genome assemblies based on 454 or IonTorrent sequencing include elevated numbers of single-base insertions and deletions owing to inaccurate sequencing (Shao et al. 2013; Zhang et al. 2015). Including such genomes in an analysis reduces the quality of the pangenome for all state-of-the-art pipelines. However, PEPPAN simply scores genes disrupted by artificial indels as frameshifts, making such inaccurate genomes easier to identify.

Finally, it is worth noting that the current similarity-based internal annotation algorithm implemented in PEPPAN is optimized for prokaryotes and does not work for eukaryotic genomes, where multiple exons of a gene can be separated by introns of >10 kb. Apart from this limitation, however, the other technological advantages in PEPPAN will also work on eukaryotic genomes. PEPPAN could therefore be extended for use on eukaryotes with collaboration from experts in eukaryotic genomics.

### Relevance to MLST schemes

Alikhan et al. (2018) described a pangenome for all of *Salmonella* based on 537 genomes that had been derived by a precursor of PEPPAN in 2015. That pangenome was used to develop a wgMLST scheme of 21,065 loci and a cgMLST scheme of 3002 genes. The same publication also described a reference set of 926 genomes that represented the diversity of almost 120,000 *Salmonella* genomes on the basis of rMLST. After completion of this paper, we became aware of a new publication (Park and Andam 2020) that used Roary to calculate a pangenome of 84,041 *S. enterica* genes and 2085 soft core genes from those 926 representative genomes after reannotation with PROKKA. Such applications of Roary are strongly discouraged by its documentation, which recommends against using Roary on diverse groups of organisms such as all *Salmonella.* We ran PEPPAN on the same 926 representative genomes. The resulting pangenome contained 30,000 fewer pan genes and 1200 more soft core genes than the calculations by Park and Andam (Supplemental Table S6), confirming that Roary struggled with this task. The high resolution and continued reliability that EnteroBase offers in downstream analyses of phylogenetic relationships between genomes are in part owing to the accurate, smaller pangenome and larger core genome that were calculated by PEPPAN. The analyses presented here identified a reliable relaxed soft core genome consisting of 292 single-copy genes for *Streptococcus*, which is currently being used to establish an EnteroBase database for this diverse genus.

### Pangenomes depend on sample size

Early analyses of pangenomes were based on small numbers of genomic sequences (Tettelin et al. 2005), resulting in the conclusion based on 12 genomes that the pangenome of *Streptococcus pyogenes* was closed (Tettelin et al. 2008). The same publication concluded that the pangenome of *Streptococcus pneumoniae* was open and would continue to expand indefinitely. However, a subsequent study of 44 genomes concluded that the pangenome of *S. pneumoniae* was also closed (Donati et al. 2010). It is only very recently that large numbers of bacterial genomes are available for analysis and that pipelines exist that can handle such large numbers.

We calculated a pangenome from 3052 *Streptococcus* genomes that represent the genomic diversity of 14,115 draft and complete genomes. Our pangenome contains 39,042 genes, is open, and will continue to expand at a rate of 4.4 genes per novel genome. This rate of expansion is 14-fold slower than the original calculations of a pangenome from 138 genomes (Gao et al. 2014). We also calculated pangenomes and their expected growth rates for the 29 most common ANI95% clusters within *Streptococcus* (Supplemental Text 4). All pangenomes were open, with the single exception of MG_41 (*Streptococcus sobrinus*). These inconsistencies with prior analyses suggest that pangenome status may be strongly dependent on the number of genomes investigated, sampling strategies used to identify representative genomes, and possibly on pangenome pipelines.

### Taxonomic insights

It has been clear since 2004 that the strict core genome of all prokaryotes is extremely small. Only 14–30 genes were present in all of 147 diverse genomes (Charlebois and Doolittle 2004), and almost all of those genes encoded ribosomal proteins (Weiss et al. 2018). However, it was still unexpected that the strict core genome would be this small for a large collection of *Streptococcus* genomes. We only found 182 strict core genes in the representative set of 3052 genomes, and each of these was absent or incomplete in one or more of the entire set of 14,115 genomes. We therefore recommend using phylogenies based on sequence variation within a relaxed core complement of genes and/or presence/absence of accessory genes for an overview of the phylogenetic relationships of an entire genus instead of relying only on strict core genes. PEPPAN_parser can calculate such phylogenies from the PEPPAN outputs.

These observations may also be relevant in respect to the concept of universal genes. FetchMG (Kultima et al. 2012) identifies the presence of genes by a very relaxed cutoff because it uses the alignment score of CDSs according to an HMM model of the corresponding protein domain. FetchMG searches for 40 supposedly universal core CDSs that are present across all prokaryotes (Mende et al. 2013). This raises the questions of why some of the 3052 *Streptococcus* genomes only contained 38 of these genes according to FetchMG (Supplemental Table S2B) and why FetchMG only found 14 that were present in all those genomes (Supplemental Table S2C). PEPPAN is stricter in its definition of strict core CDSs, because it recognizes pseudogenes and excludes them from the calls of CDSs. PEPPAN only found five strict core CDSs in all 3052 genomes (Table 1), even fewer than FetchMG. Each of these findings seem to be incompatible with a minimum of 40 universal genes for any living organism. However, previous analyses have already indicated that only 82% of genomes contain all 40 universal genes (Mende et al. 2013). Second, PEPPAN estimated the number of strict core genes including pseudogenes as 182 over all 3052 genomes (Table 1), and these included the same 14 as had been found by FetchMG. The absence of the other 26 universal genes might relate to random gaps that occur in draft genomes and that artificially resemble missing genes. Alternatively, they may not be universal.

As previously noted by others (Kilian et al. 2008; Jensen and Kilian 2012; Jensen et al. 2013, 2016; Kilian and Tettelin 2019), the taxonomies of multiple *Streptococcus* genomes are misclassified in RefSeq (Supplemental Fig. S3). Misclassification has been ongoing for decades (Kikuchi et al. 1995) owing to the phenotypic heterogeneity of this species. The Mitis group is particularly heterogeneous (Kilian et al. 2008; Jensen et al. 2016) and difficult to study (Kilian and Tettelin 2019; Velsko et al. 2019). Similar problems also apply to other bacterial genera such as *Pseudomonas* (Gomila et al. 2015) and *Aeromonas* (Beaz-Hidalgo et al. 2015). The results presented here defined 223 ANI95% clusters that are consistent by independent phylogenetic approaches based on both cgMLST and gene presence. It has been suggested that bacterial diversity does not delineate species clusters owing to extensive HGT (Doolittle and Papke 2006). Our results, instead, revealed congruent clusters between the accessory genome and the core genome at the ANI95% level in *Streptococcus.* Similar congruent clusters have been reported in the *Streptomycetaceae* (Wright and Baum 2018) and we suspect that they will also occur in other genera. Thus, approaches such as those described here may provide a framework for improving future taxonomic assignments. Finally, the test case of *Streptococcus* illustrates the power of PEPPAN, which can now be used for defining the pangenomes of other diverse genera.

## Methods

### *S. enterica* genomes

We downloaded the assembly_summary_genbank.txt table and the assembly_summary_refseq.txt table from NCBI on May 30, 2019 (ftp://ftp.ncbi.nlm.nih.gov/genomes/ASSEMBLY_REPORTS/). The first table summarizes all genomes uploaded into GenBank by their original investigators, and the second summarizes all the genomes in RefSeq. We used these tables as a source of the FTP links for each of the accession codes listed by Nuccio and Bäumler (2014) for genomic sequences of 15 *S. enterica* genomes. These 15 genomes were also annotated ab initio with PROKKA 1.12 (Seemann 2014). Nuccio and Bäumler excluded some “unreliable” short genes from their manual recuration. To exclude these genes in our analyses as well, the genomic coordinates of each gene in each of the three annotations (Submitter, RefSeq, PROKKA) were compared with the coordinates of “reliable genes” in Supplemental Table S1 of Nuccio and Bäumler. Only genes with coordinates overlapping those of a reliable gene by ≥90% were used here for further comparisons.

### Preparation of simulated data sets

All simulated data sets were generated using SimPan (Supplemental Text 2) with the input parameters “‐‐genomeNum 15 ‐‐aveSize 4500 ‐‐pBackbone 4000 ‐‐nMobile 10000 ‐‐nCore 3621 ‐‐pBackbone 0.05 ‐‐pMobile 0.40 ‐‐rec 0.1”. Data sets a through e were generated with the additional parameters: (a) “‐‐idenOrtholog 0.9 ‐‐idenParalog 0.6”; (b) “‐‐idenOrtholog 0.95 ‐‐idenParalog 0.6”; (c) “‐‐idenOrtholog 0.98 ‐‐idenParalog 0.6”; (d) “‐‐idenOrtholog 0.98 ‐‐idenParalog 0.8”; (e) “‐‐idenOrtholog 0.98 ‐‐idenParalog 0.9”. Ten other sets of simulated genomes that were used to evaluate running times were generated with the same parameters as data set c but with the additional parameter “‐‐genomeNum xxx”, where xxx ranged from 20 to 200 by steps of 20.

### Pangenome pipelines

The following versions of the individual pipelines and command lines were used for all benchmark data sets.

1. PEPPAN with a Git HEAD of f721513 was run in the Python 3.6 environment as
  python PEPPA.py -t 4 -p PEPPAN ‐‐pseudogene 0.9 ‐‐min_cds 45 *.gff
2. Roary 3.6.0+dfsg-4 was installed as a Ubuntu APT package and run as
  roary -p 4 -o roary -f roary -i <identity>-s -v -y *.gff
  Three runs of Roary were performed for each data set with the additional parameters “-i 50,” “-i 80,” or “-i 95.” The data reported here are from the runs with the parameter “-i 80” because that consistently yielded the best ARI values.
3. PIRATE with a Git HEAD of effc522 was downloaded from GitHub (https://github.com/SionBayliss/PIRATE) and run as
  PIRATE -i . -o PIRATE -s <steps> -t 4 -k “‐‐diamond”
  Three runs of PIRATE were performed for each data set with the additional parameters “-s 50,60,70,80,90,95,98,” “-s 80,90,95,98,” or “-s 95,98.” We report the data generated with “-s 80,90,95,98,” which had the greatest ARI value, except for simulated data set e, in which “-s 95,98” had the greatest ARI.
4. panX v1.6.0 was downloaded from GitHub (https://github.com/neherlab/pan-genome-analysis/releases) and run in the Python 2.7 environment as
  panX.py ‐‐folder_name panX ‐‐species_name panX ‐‐threads 4 ‐‐diamond_identity 80 ‐‐simple_tree ‐‐store_locus_tag
5. OrthoMCL v2.0.9 was downloaded from https://orthomcl.org/ and run in multiple steps as described at https://currentprotocols.onlinelibrary.wiley.com/doi/full/10.1002/0471250953.bi0612s35.

### Generating ANI95% clusters of *Streptococcus* genomes

A summary table of all genomes deposited in RefSeq was downloaded on June 20, 2019 (see *S. enterica* genomes above); 14,115 bacterial records that contained “Streptococcus” in the “organism_name” field were extracted from the table (Supplemental Table S7), and the files for each record were downloaded as described above. MASH (Ondov et al. 2016) was used to measure the pairwise distances between the genomes with parameters of “-k 19 –s 10000.” The resulting matrix was used to cluster *Streptococcus* genomes with the AgglomerativeClustering function in the scikit-learn package (Pedregosa et al. 2011), with parameters linkage = single and distance_threshold = 0.002. The function generated 3170 clusters. The genome with the greatest N50 value within each cluster was chosen as its representative genome. Each representative genome was subjected to quality evaluation according to three criteria: (1) carries at least 38 of the 40 single-copy essential genes according to fetchMG (Kultima et al. 2012), (2) is assigned to *Streptococcus* genus by the “Identify species” function in rmlst.org (Jolley et al. 2012), and (3) has an N50 value ≥10 kb. One hundred eighteen genomes failed these criteria and were discarded (Supplemental Table S2B), leaving a data set of 3052 high-quality genomes (Supplemental Table S2A) that represents the entire taxonomic diversity of *Streptococcus*. Pairwise ANI values were calculated from the 3052 representative genomes with FastANI v1.2 (Jain et al. 2018), and these genomes were grouped into ANI95% clusters using the AgglomerativeClustering function with linkage = single and distance_threshold=0.05.

### Software availability

Source code for PEPPAN is accessible at GitHub (https://github.com/zheminzhou/PEPPAN) and as Supplemental Code S1. Source code for SimPan is accessible at GitHub (https://github.com/zheminzhou/SimPan) and as Supplemental Code S2.

## Competing interest statement

The authors declare no competing interests.

## Supplementary Material

Supplemental Material

## References

[GR260828ZHOC1] Abby SS, Tannier E, Gouy M, Daubin V. 2012 Lateral gene transfer as a support for the tree of life. Proc Natl Acad Sci 109: 4962–4967. 10.1073/pnas.1116871109 22416123 PMC3323970

[GR260828ZHOC2] Achtman M, Zhou Z. 2020 Metagenomics of the modern and historical human oral microbiome with phylogenetic studies on *Streptococcus mutans* and *Streptococcus sobrinus*. Phil Trans R Soc B **375:** 20190573 10.1098/rstb.2019.0573 33012228 PMC7702799

[GR260828ZHOC3] Alikhan N-F, Zhou Z, Sergeant MJ, Achtman M. 2018 A genomic overview of the population structure of *Salmonella*. PLoS Genet 14: e1007261 10.1371/journal.pgen.1007261 29621240 PMC5886390

[GR260828ZHOC4] Altenhoff AM, Glover NM, Dessimoz C. 2019 Inferring orthology and paralogy In Evolutionary genomics: statistical and computational methods (ed. Anisimova M), pp. 149–175. Springer New York, New York. 10.1007/978-1-4939-9074-0_5 31278664

[GR260828ZHOC5] Altschul SF, Gish W, Miller W, Myers EW, Lipman DJ. 1990 Basic local alignment search tool. J Mol Biol 215: 403–410. 10.1016/S0022-2836(05)80360-2 2231712

[GR260828ZHOC6] Banaszkiewicz S, Calland JK, Mourkas E, Sheppard SK, Pascoe B, Bania J. 2019 Genetic diversity of composite enterotoxigenic *Staphylococcus epidermidis* pathogenicity islands. Genome Biol Evol 11: 3498–3509. 10.1093/gbe/evz259 31769803 PMC6931896

[GR260828ZHOC7] Bayliss SC, Thorpe HA, Coyle NM, Sheppard SK, Feil EJ. 2019 PIRATE: a fast and scalable pangenomics toolbox for clustering diverged orthologues in bacteria. Gigascience 8: giz119 10.1093/gigascience/giz119 31598686 PMC6785682

[GR260828ZHOC8] Beaz-Hidalgo R, Hossain MJ, Liles MR, Figueras MJ. 2015 Strategies to avoid wrongly labelled genomes using as example the detected wrong taxonomic affiliation for *Aeromonas* genomes in the GenBank database. PLoS One 10: e0115813 10.1371/journal.pone.0115813 25607802 PMC4301921

[GR260828ZHOC9] Brown T, Didelot X, Wilson DJ, De MN. 2016 SimBac: simulation of whole bacterial genomes with homologous recombination. Microb Genom 2: e000044 10.1099/mgen.0.000044 PMC5049688 27713837

[GR260828ZHOC10] Buchfink B, Xie C, Huson DH. 2015 Fast and sensitive protein alignment using DIAMOND. Nat Methods 12: 59–60. 10.1038/nmeth.3176 25402007

[GR260828ZHOC11] Charlebois RL, Doolittle WF. 2004 Computing prokaryotic gene ubiquity: rescuing the core from extinction. Genome Res 14: 2469–2477. 10.1101/gr.3024704 15574825 PMC534671

[GR260828ZHOC12] Chewapreecha C, Harris SR, Croucher NJ, Turner C, Marttinen P, Cheng L, Pessia A, Aanensen DM, Mather AE, Page AJ, 2014 Dense genomic sampling identifies highways of pneumococcal recombination. Nature Genet 46: 305–309. 10.1038/ng.2895 24509479 PMC3970364

[GR260828ZHOC13] Croucher NJ, Coupland PG, Stevenson AE, Callendrello A, Bentley SD, Hanage WP. 2014 Diversification of bacterial genome content through distinct mechanisms over different timescales. Nat Commun 5: 5471 10.1038/ncomms6471 25407023 PMC4263131

[GR260828ZHOC14] Daubin V, Ochman H. 2004 Bacterial genomes as new gene homes: the genealogy of ORFans in *E. coli*. Genome Res 14: 1036–1042. 10.1101/gr.2231904 15173110 PMC419781

[GR260828ZHOC15] Dekker JP, Lau AF. 2016 An update on the *Streptococcus bovis* group: classification, identification, and disease associations. J Clin Microbiol 54: 1694–1699. 10.1128/JCM.02977-15 26912760 PMC4922088

[GR260828ZHOC16] Denton JF, Lugo-Martinez J, Tucker AE, Schrider DR, Warren WC, Hahn MW. 2014 Extensive error in the number of genes inferred from draft genome assemblies. PLoS Comput Biol 10: e1003998 10.1371/journal.pcbi.1003998 25474019 PMC4256071

[GR260828ZHOC17] Ding W, Baumdicker F, Neher RA. 2018 panX: pan-genome analysis and exploration. Nucleic Acids Res 46: e5 10.1093/nar/gkx977 29077859 PMC5758898

[GR260828ZHOC18] Donati C, Hiller NL, Tettelin H, Muzzi A, Croucher NJ, Angiuoli SV, Oggioni M, Dunning Hotopp JC, Hu FZ, Riley DR, 2010 Structure and dynamics of the pan-genome of *Streptococcus pneumoniae* and closely related species. Genome Biol 11: R107 10.1186/gb-2010-11-10-r107 21034474 PMC3218663

[GR260828ZHOC19] Doolittle WF, Papke RT. 2006 Genomics and the bacterial species problem. Genome Biol 7: 116 10.1186/gb-2006-7-9-116 17020593 PMC1794555

[GR260828ZHOC20] Enright AJ, Van DS, Ouzounis CA. 2002 An efficient algorithm for large-scale detection of protein families. Nucleic Acids Res 30: 1575–1584. 10.1093/nar/30.7.1575 11917018 PMC101833

[GR260828ZHOC21] Fleischmann RD, Adams MD, White O, Clayton RA, Kirkness EF, Kerlavage AR, Bult CJ, Tomb JF, Dougherty BA, Merrick JM, 1995 Whole-genome random sequencing and assembly of *Haemophilus influenzae* Rd. Science 269: 496–512. 10.1126/science.7542800 7542800

[GR260828ZHOC22] Fletcher W, Yang Z. 2009 INDELible: a flexible simulator of biological sequence evolution. Mol Biol Evol 26: 1879–1888. 10.1093/molbev/msp098 19423664 PMC2712615

[GR260828ZHOC23] Frentrup M, Zhou Z, Steglich M, Meier-Kolthoff JP, Göker M, Riedel T, Bunk B, Spröer C, Overmann J, Blaschitz M, 2020 A publicly accessible database for *Clostridioides difficile* genome sequences supports tracing of transmission chains and epidemics. Microbial Genomics 6: mgen.0.000410 10.1099/mgen.0.000410 PMC7641423 32726198

[GR260828ZHOC24] Gao X-Y, Zhi XY, Li HW, Klenk HP, Li WJ. 2014 Comparative genomics of the bacterial genus *Streptococcus* illuminates evolutionary implications of species groups. PLoS One 9: e101229 10.1371/journal.pone.0101229 24977706 PMC4076318

[GR260828ZHOC25] Gomila M, Pena A, Mulet M, Lalucat J, Garcia-Valdes E. 2015 Phylogenomics and systematics in *Pseudomonas*. Front Microbiol 6: 214 10.3389/fmicb.2015.00214 26074881 PMC4447124

[GR260828ZHOC26] Goodhead I, Darby AC. 2015 Taking the pseudo out of pseudogenes. Curr Opin Microbiol 23: 102–109. 10.1016/j.mib.2014.11.012 25461580

[GR260828ZHOC27] Haft DH, DiCuccio M, Badretdin A, Brover V, Chetvernin V, O'Neill K, Li W, Chitsaz F, Derbyshire MK, Gonzales NR, 2018 Refseq: an update on prokaryotic genome annotation and curation. Nucleic Acids Res 46: D851–D860. 10.1093/nar/gkx1068 29112715 PMC5753331

[GR260828ZHOC28] Hogg JS, Hu FZ, Janto B, Boissy R, Hayes J, Keefe R, Post JC, Ehrlich GD. 2007 Characterization and modeling of the *Haemophilus influenzae* core and supragenomes based on the complete genomic sequences of Rd and 12 clinical nontypeable strains. Genome Biol 8: R103 10.1186/gb-2007-8-6-r103 17550610 PMC2394751

[GR260828ZHOC29] Holt KE, Parkhill J, Mazzoni CJ, Roumagnac P, Weill F-X, Goodhead I, Rance R, Baker S, Maskell D, Wain J, 2008 High-throughput sequencing provides insights into genome variation and evolution in *Salmonella* typhi. Nature Genet 40: 987–993. 10.1038/ng.195 18660809 PMC2652037

[GR260828ZHOC30] Jain C, Rodriguez-R LM, Phillippy AM, Konstantinidis KT, Aluru S. 2018 High throughput ANI analysis of 90K prokaryotic genomes reveals clear species boundaries. Nat Commun 9: 5114 10.1038/s41467-018-07641-9 30504855 PMC6269478

[GR260828ZHOC31] Jensen A, Kilian M. 2012 Delineation of *Streptococcus dysgalactiae*, its subspecies, and its clinical and phylogenetic relationship to *Streptococcus pyogenes*. J Clin Microbiol 50: 113–126. 10.1128/JCM.05900-11 22075580 PMC3256718

[GR260828ZHOC32] Jensen A, Hoshino T, Kilian M. 2013 Taxonomy of the Anginosus group of the genus *Streptococcus* and description of *Streptococcus anginosus* subsp. *Whileyi* subsp. nov. and *Streptococcus constellatus* subsp. *viborgensis* subsp. nov. Int J Syst Evol Microbiol 63: 2506–2519. 10.1099/ijs.0.043232-0 23223817

[GR260828ZHOC33] Jensen A, Scholz CF, Kilian M. 2016 Re-evaluation of the taxonomy of the mitis group of the genus *Streptococcus* based on whole genome phylogenetic analyses, and proposed reclassification of *Streptococcus dentisani* as *Streptococcus oralis* subsp. *dentisani* comb. nov., *Streptococcus tigurinus* as *Streptococcus oralis* subsp. *tigurinus* comb. nov., and *Streptococcus oligofermentans* as a later synonym of *Streptococcus cristatus*. Int J Syst Evol Microbiol 66: 4803–4820. 10.1099/ijsem.0.001433 27534397

[GR260828ZHOC34] Jolley KA, Bliss CM, Bennett JS, Bratcher HB, Brehony C, Colles FM, Wimalarathna H, Harrison OB, Sheppard SK, Cody AJ, 2012 Ribosomal multilocus sequence typing: universal characterization of bacteria from domain to strain. Microbiology 158: 1005–1015. 10.1099/mic.0.055459-0 22282518 PMC3492749

[GR260828ZHOC35] Katoh K, Standley DM. 2013 MAFFT multiple sequence alignment software version 7: improvements in performance and usability. Mol Biol Evol 30: 772–780. 10.1093/molbev/mst010 23329690 PMC3603318

[GR260828ZHOC36] Kikuchi K, Enari T, Totsuka K, Shimizu K. 1995 Comparison of phenotypic characteristics, DNA-DNA hybridization results, and results with a commercial rapid biochemical and enzymatic reaction system for identification of viridans group streptococci. J Clin Microbiol 33: 1215–1222. 10.1128/JCM.33.5.1215-1222.1995 7615731 PMC228134

[GR260828ZHOC37] Kilian M, Tettelin H. 2019 Identification of virulence-associated properties by comparative genome analysis of *Streptococcus pneumoniae, S. pseudopneumoniae, S. mitis*, three *S. oralis* subspecies, and *S. infantis*. mBio 10: e01985-19 10.1128/mBio.01985-19 31481387 PMC6722419

[GR260828ZHOC38] Kilian M, Poulsen K, Blomqvist T, Håvarstein LS, Bek-Thomsen M, Tettelin H, Sørensen UB. 2008 Evolution of *Streptococcus pneumoniae* and its close commensal relatives. PLoS One 3: e2683 10.1371/journal.pone.0002683 18628950 PMC2444020

[GR260828ZHOC39] Konstantinidis KT, Rosselló-Móra R, Amann R. 2017 Uncultivated microbes in need of their own taxonomy. ISME J 11: 2399–2406. 10.1038/ismej.2017.113 28731467 PMC5649169

[GR260828ZHOC40] Kultima JR, Sunagawa S, Li J, Chen W, Chen H, Mende DR, Arumugam M, Pan Q, Liu B, Qin J, 2012 MOCAT: a metagenomics assembly and gene prediction toolkit. PLoS One 7: e47656 10.1371/journal.pone.0047656 23082188 PMC3474746

[GR260828ZHOC41] Laing C, Buchanan C, Taboada EN, Zhang Y, Kropinski A, Villegas A, Thomas JE, Gannon VP. 2010 Pan-genome sequence analysis using panseq: an online tool for the rapid analysis of core and accessory genomic regions. BMC Bioinformatics 11: 461 10.1186/1471-2105-11-461 20843356 PMC2949892

[GR260828ZHOC42] Lerat E, Ochman H. 2005 Recognizing the pseudogenes in bacterial genomes. Nucleic Acids Res 33: 3125–3132. 10.1093/nar/gki631 15933207 PMC1142405

[GR260828ZHOC43] Li L, Stoeckert CJ Jr., Roos DS. 2003 OrthoMCL: identification of ortholog groups for eukaryotic genomes. Genome Res 13: 2178–2189. 10.1101/gr.1224503 12952885 PMC403725

[GR260828ZHOC44] Mende DR, Sunagawa S, Zeller G, Bork P. 2013 Accurate and universal delineation of prokaryotic species. Nat Methods 10: 881–884. 10.1038/nmeth.2575 23892899

[GR260828ZHOC45] Moura A, Criscuolo A, Pouseele H, Maury MM, Leclercq A, Tarr C, Björkman JT, Dallman T, Reimer A, Enouf V, 2017 Whole genome-based population biology and epidemiological surveillance of *Listeria monocytogenes*. Nat Microbiol 2: 16185 10.1038/nmicrobiol.2016.185 PMC8903085 27723724

[GR260828ZHOC46] Nuccio SP, Bäumler AJ. 2014 Comparative analysis of *salmonella* genomes identifies a metabolic network for escalating growth in the inflamed gut. mBio 5: e00929-14 10.1128/mBio.00929-14 24643865 PMC3967523

[GR260828ZHOC47] Ondov BD, Treangen TJ, Melsted P, Mallonee AB, Bergman NH, Koren S, Phillippy AM. 2016 Mash: fast genome and metagenome distance estimation using minHash. Genome Biol 17: 132 10.1186/s13059-016-0997-x 27323842 PMC4915045

[GR260828ZHOC48] Page AJ, Cummins CA, Hunt M, Wong VK, Reuter S, Holden MT, Fookes M, Falush D, Keane JA, Parkhill J. 2015 Roary: rapid large-scale prokaryote pan genome analysis. Bioinformatics 31: 3691–3693. 10.1093/bioinformatics/btv421 26198102 PMC4817141

[GR260828ZHOC49] Park CJ, Andam CP. 2020 Distinct but intertwined evolutionary histories of multiple *Salmonella enterica* subspecies. mSystems 5: e00515-19 10.1128/mSystems.00515-19 PMC6967386 31937675

[GR260828ZHOC50] Parkhill J, Dougan G, James KD, Thomson NR, Pickard D, Wain J, Churcher C, Mungall KL, Bentley SD, Holden MT, 2001 Complete genome sequence of a multiple drug resistant *Salmonella enterica* serovar typhi CT18. Nature 413: 848–852. 10.1038/35101607 11677608

[GR260828ZHOC51] Pedregosa F, Vatoquaux G, Gramfort A, Michel V, Thirion B, Grisel O, Blondel M, Prettenhofer P, Weiss R, Dubourg V, 2011 Scikit-learn: machine learning in Python. J Mach Learn Res 12: 2825–2830.

[GR260828ZHOC52] Price MN, Dehal PS, Arkin AP. 2010 Fasttree 2: approximately maximum-likelihood trees for large alignments. PLoS One 5: e9490 10.1371/journal.pone.0009490 20224823 PMC2835736

[GR260828ZHOC53] Rand WM. 1971 Objective criteria for the evaluation of clustering methods. J Amer Statist Assoc 66: 846–850. 10.1080/01621459.1971.10482356

[GR260828ZHOC54] Salzberg SL. 2019 Next-generation genome annotation: We still struggle to get it right. Genome Biol 20: 92 10.1186/s13059-019-1715-2 31097009 PMC6521345

[GR260828ZHOC55] Sanaa M, Pouillot R, Vega FG, Strain E, Van Doren JM. 2019 Genomegraphr: a user-friendly open-source web application for foodborne pathogen whole genome sequencing data integration, analysis, and visualization. PLoS One 14: e0213039 10.1371/journal.pone.0213039 30818354 PMC6394949

[GR260828ZHOC56] Seemann T. 2014 Prokka: rapid prokaryotic genome annotation. Bioinformatics 30: 2068–2069. 10.1093/bioinformatics/btu153 24642063

[GR260828ZHOC57] Shao W, Boltz VF, Spindler JE, Kearney MF, Maldarelli F, Mellors JW, Stewart C, Volfovsky N, Levitsky A, Stephens RM, 2013 Analysis of 454 sequencing error rate, error sources, and artifact recombination for detection of Low-frequency drug resistance mutations in HIV-1 DNA. Retrovirology 10: 18 10.1186/1742-4690-10-18 23402264 PMC3599717

[GR260828ZHOC58] Simonsen M, Mailund T, Pedersen CNS. 2011 Inference of large phylogenies using neighbour-joining In Biomedical engineering systems and technologies, BIOSTEC 2010: Communications in Computer and Information Science (ed. Fred A, ), Vol. 127, pp. 334–344. Springer Verlag, Berlin.

[GR260828ZHOC59] Steinegger M, Söding J. 2017 MMseqs2 enables sensitive protein sequence searching for the analysis of massive data sets. Nat Biotechnol 35: 1026–1028. 10.1038/nbt.3988 29035372

[GR260828ZHOC60] Steinegger M, Söding J. 2018 Clustering huge protein sequence sets in linear time. Nat Commun 9: 2542 10.1038/s41467-018-04964-5 29959318 PMC6026198

[GR260828ZHOC61] Szöllösi GJ, Boussau B, Abby SS, Tannier E, Daubin V. 2012 Phylogenetic modeling of lateral gene transfer reconstructs the pattern and relative timing of speciations. Proc Natl Acad Sci 109: 17513–17518. 10.1073/pnas.1202997109 23043116 PMC3491530

[GR260828ZHOC62] Tatusova T, DiCuccio M, Badretdin A, Chetvernin V, Nawrocki EP, Zaslavsky L, Lomsadze A, Pruitt KD, Borodovsky M, Ostell J. 2016 NCBI prokaryotic genome annotation pipeline. Nucleic Acids Res 44: 6614–6624. 10.1093/nar/gkw569 27342282 PMC5001611

[GR260828ZHOC63] Tettelin H, Masignani V, Cieslewicz MJ, Donati C, Medini D, Ward NL, Angiuoli SV, Crabtree J, Jones AL, Durkin AS, 2005 Genome analysis of multiple pathogenic isolates of *Streptococcus agalactiae*: implications for the microbial “pan-genome”. Proc Natl Acad Sci 102: 13950–13955. 10.1073/pnas.0506758102 16172379 PMC1216834

[GR260828ZHOC64] Tettelin H, Riley D, Cattuto C, Medini D. 2008 Comparative genomics: the bacterial pan-genome. Curr Opin Microbiol 11: 472–477. 10.1016/j.mib.2008.09.006 19086349

[GR260828ZHOC65] Velsko IM, Chakraborty B, Nascimento MM, Burne RA, Richards VP. 2018 Species designations belie phenotypic and genotypic heterogeneity in oral streptococci. mSystems 3: e00158-18 10.1128/mSystems.00158-18 PMC6299155 30574560

[GR260828ZHOC66] Velsko IM, Perez MS, Richards VP. 2019 Resolving phylogenetic relationships for *Streptococcus mitis* and *Streptococcus oralis* through core- and pan-genome analyses. Genome Biol Evol 11: 1077–1087. 10.1093/gbe/evz049 30847473 PMC6461889

[GR260828ZHOC67] Vernikos G, Medini D, Riley DR, Tettelin H. 2015 Ten years of pan-genome analyses. Curr Opin Microbiol 23: 148–154. 10.1016/j.mib.2014.11.016 25483351

[GR260828ZHOC68] Weiss MC, Preiner M, Xavier JC, Zimorski V, Martin WF. 2018 The last universal common ancestor between ancient earth chemistry and the onset of genetics. PLoS Genet 14: e1007518 10.1371/journal.pgen.1007518 30114187 PMC6095482

[GR260828ZHOC69] Wozniak M, Wong L, Tiuryn J. 2014 eCAMBer: efficient support for large-scale comparative analysis of multiple bacterial strains. BMC Bioinformatics 15: 65 10.1186/1471-2105-15-65 24597904 PMC4023553

[GR260828ZHOC70] Wright ES, Baum DA. 2018 Exclusivity offers a sound yet practical species criterion for bacteria despite abundant gene flow. BMC Genomics 19: 724 10.1186/s12864-018-5099-6 30285620 PMC6171291

[GR260828ZHOC71] Zhang B, Penton CR, Xue C, Wang Q, Zheng T, Tiedje JM. 2015 Evaluation of the Ion Torrent personal genome machine for gene-targeted studies using amplicons of the nitrogenase gene *nifH*. Appl Environ Microbiol 81: 4536–4545. 10.1128/AEM.00111-15 25911484 PMC4475867

[GR260828ZHOC72] Zhao Y, Wu J, Yang J, Sun S, Xiao J, Yu J. 2012 PGAP: pan-genomes analysis pipeline. Bioinformatics 28: 416–418. 10.1093/bioinformatics/btr655 22130594 PMC3268234

[GR260828ZHOC73] Zhou Z, McCann A, Weill F-X, Blin C, Nair S, Wain J, Dougan G, Achtman M. 2014 Transient Darwinian selection in *Salmonella enterica* serovar Paratyphi A during 450 years of global spread of enteric fever. Proc Natl Acad Sci 111: 12199–12204. 10.1073/pnas.1411012111 25092320 PMC4143038

[GR260828ZHOC74] Zhou Z, Alikhan N-F, Sergeant MJ, Luhmann N, Vaz C, Francisco AP, Carriço JA, Achtman M. 2018a Grapetree: visualization of core genomic relationships among 100,000 bacterial pathogens. Genome Res 28: 1395–1404. 10.1101/gr.232397.117 30049790 PMC6120633

[GR260828ZHOC75] Zhou Z, Lundstrøm I, Tran-Dien A, Duchêne S, Alikhan N-F, Sergeant MJ, Langridge G, Fokatis AK, Nair S, Stenøien HK, 2018b Pan-genome analysis of ancient and modern *Salmonella enterica* demonstrates genomic stability of the invasive para C lineage for millennia. Curr Biol 28: 2420–2428.e10. 10.1016/j.cub.2018.05.058 30033331 PMC6089836

[GR260828ZHOC76] Zhou Z, Alikhan N-F, Mohamed K, Fan Y, Agama Study Group, Achtman M. 2020 The EnteroBase user's guide, with case studies on *Salmonella* transmissions, *Yersinia pestis* phylogeny, and *Escherichia* core genomic diversity. Genome Res 30: 138–152. 10.1101/gr.251678.119 31809257 PMC6961584

