# Supplementary material for: Accurate reconstruction of bacterial pan- and core genomes with PEPPAN
Source: Genome Res. 2020 Nov;30(11):1667–79. doi: 10.1101/gr.260828.120 (PMC7605250; doi:10.1101/gr.260828.120)
Supplement: Supplemental Material [file supp_30_11_1667__index.html]

Accurate reconstruction of bacterial pan- and core genomes with PEPPAN — Supplemental Material 

# Accurate reconstruction of bacterial pan- and core genomes with PEPPAN

## Supplemental Material

- Supplemental\_Texts.pdf
- Supplemental\_Fig\_S1.pdf
- Supplemental\_Fig\_S2.pdf
- Supplemental\_Fig\_S3.pdf
- Supplemental\_Table\_S1.pdf
- Supplemental\_Table\_S3.xlsx
- Supplemental\_Table\_S4.xlsx
- Supplemental\_Table\_S5.pdf
- Supplemental\_Table\_S6.pdf
- Supplemental\_Table\_S7.xlsx
- Supplemental\_Code\_S1.zip
- Supplemental\_Code\_S2.zip
- Supplemental\_Table\_S2\_revised.xlsx
