# Supplementary material for: Accurate reconstruction of bacterial pan- and core genomes with PEPPAN
Source: Genome Res. 2020 Nov;30(11):1667–79. doi: 10.1101/gr.260828.120 (PMC7605250; doi:10.1101/gr.260828.120)
Supplement: Supplemental Material [file supp_gr.260828.120_Supplemental_Fig_S2.pdf]

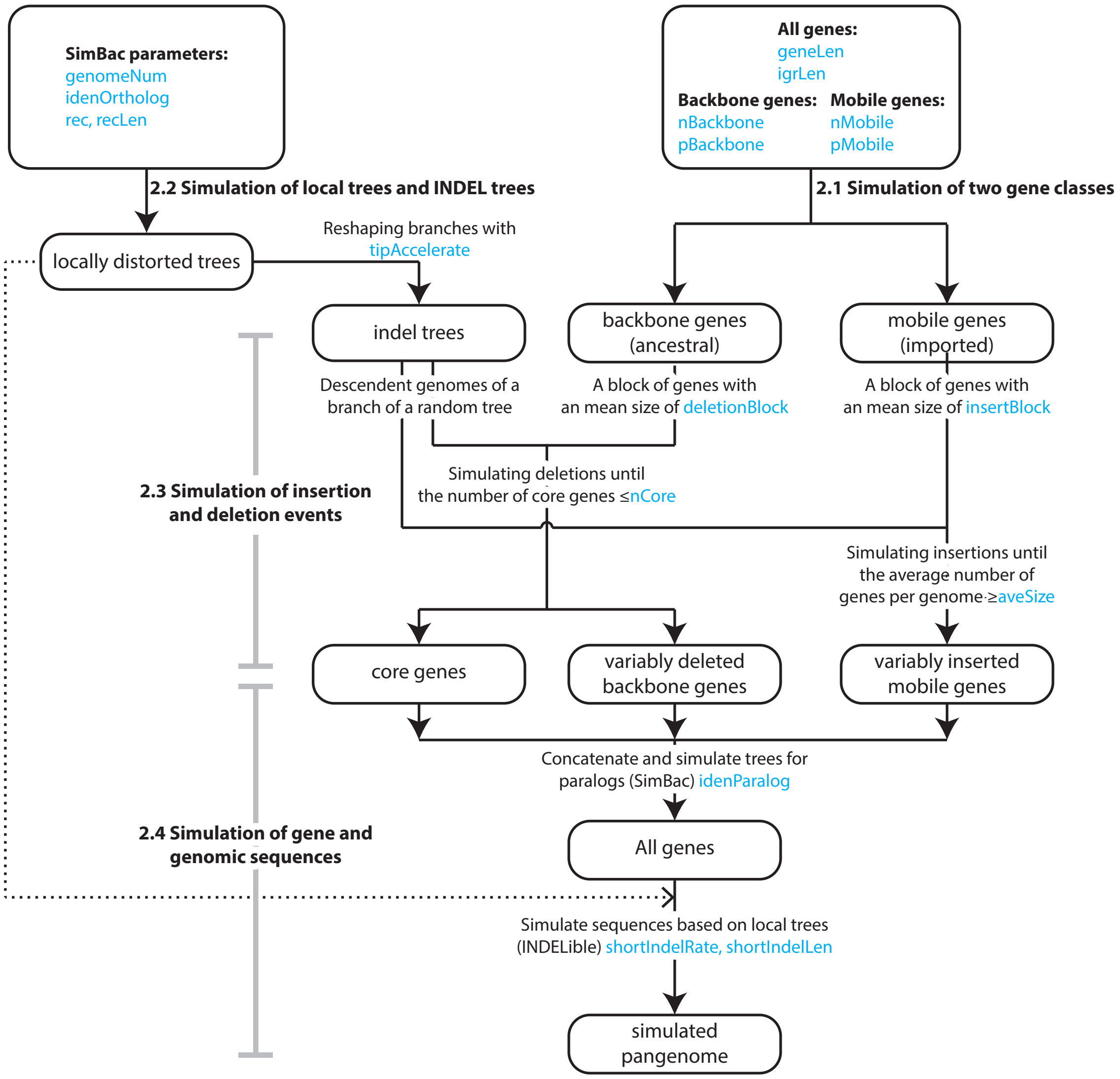

Supplemental Figure S2. The detailed workflow of SimPan. The operations initiated with 2.x and in bold fonts are sub-titles that corresponds to sub-titles in Supplemental Text 2. The words in blue are the names of the parameters to be specified by the users (Supplemental Table S1).
