# Supplementary material for: Accurate reconstruction of bacterial pan- and core genomes with PEPPAN
Source: Genome Res. 2020 Nov;30(11):1667–79. doi: 10.1101/gr.260828.120 (PMC7605250; doi:10.1101/gr.260828.120)
Supplement: Supplemental Material [file supp_gr.260828.120_Supplemental_Table_S1.pdf]

Supplemental Table S1. Available options in SimPan

Option	Description	Default
genomeNum	number of genomes in population	20
idenOrtholog	average nucleotide identities of orthologous genes	0.98
rec	expected proportion of homoplastic sites between pairs of genomes	0.05
recLen	expected length (bp) of a recombinant event	1000
geneLen	mean, minimum, and maximum gene length (bp)	900,150,6000
igrLen	mean, minimum and maximum length (bp) of intergenic regions	50,0,300
nBackbone	number of backbone genes present in the common ancestor	4000
pBackbone	proportion of paralogs in the backbone genes	0.05
nMobile	number of mobile genes	20000
pMobile	proportion of paralogs in the mobile genes	0.4
tipAccelerate	gradient by which to accelerate rate of recent gene indel events	100
deletionBlock	mean, minimum and maximum number of genes in a deletion event	3,0,30
insertBlock	mean, minimum and maximum number of genes in an insertion event	10,0,100
nCore	number of strict core genes	3500
aveSize	average number of genes per genome	4500
idenParalog	average nucleotide identities of paralogous genes	0.6
shortIndelRate	average frequency of indel events relative to mutation rates	0.01
shortIndelLen	average length (bp) of short indel events within a gene	10
