# Supplementary material for: Accurate reconstruction of bacterial pan- and core genomes with PEPPAN
Source: Genome Res. 2020 Nov;30(11):1667–79. doi: 10.1101/gr.260828.120 (PMC7605250; doi:10.1101/gr.260828.120)
Supplement: Supplemental Material [file supp_gr.260828.120_Supplemental_Table_S5.pdf]

Supplemental Table S5. *Streptococcus* pangenomes from 29 ANI 95% clusters with ≥10 genomes.

Group	Species	No. of Genomes	mean ANI%	No. of CDSs	No. of Genes	% pseudo	No. of Core genes	No. of core CDSs	No. of pan genes	α
**MG_4**	*S. agalactiae*	224	98.86	1,895	1,994	5.0	1,037	433	6,361	0.80
**MG_69**	*S. anginosus*	57	95.94	1,730	1,832	5.6	1,184	979	5,393	0.79
**MG_34**	*S. constellatus*	16	97.96	1,674	1,785	6.2	1,348	1,117	3,418	0.67
**MG_37**	*S. cristatus*	12	95.62	1,800	1,892	4.9	1,460	1,364	3,375	0.65
**MG_2**	*S. dysgalactiae*	41	98.17	1,865	1,959	4.8	1,396	1,124	4,620	0.75
**MG_114**	*S. equi*	23	97.37	1,755	1,846	4.9	1,398	1,201	3,467	0.73
**MG_8**	*S. equinus*	23	97.72	1,649	1,718	4.0	1,286	975	3,410	0.62
**MG_23**	*S. equinus*	11	96.65	1,723	1,806	4.6	1,396	1,257	3,088	0.64
**MG_29**	*S. gallolyticus*	36	96.54	1,914	2,048	6.5	1,165	836	6,206	0.76
**MG_32**	*S. gordonii*	42	96.17	1,940	2,026	4.3	1,534	1,239	4,051	0.75
**MG_101**	*S. iniae*	12	99.63	1,836	1,928	4.7	1,551	1,400	2,854	0.81
**MG_150**	*S. intermedius*	30	98.37	1,726	1,820	5.2	1,412	1,151	3,643	0.81
**MG_33**	*S. mitis*	20	95.36	1,776	1,900	6.5	1,229	929	4,335	0.74
**MG_43**	*S. mitis*	14	95.76	1,696	1,773	4.3	1,352	1,139	3,034	0.80
**MG_156**	*S. mutans*	84	98.84	1,744	1,837	5.0	1,206	914	3,385	0.89
**MG_18**	*S. oralis subsp. oralis*	54	95.19	1,774	1,849	4.1	1,323	1085	4,805	0.71
**MG_143**	*S. oralis subsp. tigurinus*	10	95.91	1,752	1,815	3.5	1,464	1,393	2,799	0.71
**MG_13**	*S. parasanguinis*	33	95.38	1,864	1,951	4.5	1,343	1,105	4,267	0.82
**MG_12**	*S. parasanguinis*	10	96.92	1,863	1,949	4.4	1,448	1,274	3,132	0.78
**MG_55**	*S. parauberis*	14	99.08	1,882	1,980	4.9	1,520	1,311	3,749	0.60
**MG_27**	*S. pneumoniae*	997	98.50	1,857	1,992	6.8	840	195	6,457	0.84
**MG_75**	*S. pseudopneumoniae*	44	97.31	1,877	2,020	7.1	1,498	1,211	3,223	0.91
**MG_1**	*S. pyogenes*	441	98.78	1,590	1,656	4.0	1,249	761	4,246	0.85
**MG_7**	*S. salivarius*	53	95.78	1,880	1,968	4.5	1,323	1,083	4,657	0.81
**MG_16**	*S. sanguinis*	47	95.41	2,135	2,212	3.5	1,641	1,460	4,549	0.87
**MG_41**	*S. sobrinus*	21	98.82	1,702	1,900	10.4	1,529	614	2,577	1.04
**MG_11**	*S. suis*	321	95.51	1,952	2,061	5.3	989	648	9,947	0.82
**MG_21**	*S. thermophilus*	33	98.80	1,491	1,711	12.9	1,273	956	3,028	0.73
**MG_66**	*S. uberis*	17	99.07	1,746	1,804	3.2	1,463	1,391	3,132	0.59
