# Supplementary material for: Accurate reconstruction of bacterial pan- and core genomes with PEPPAN
Source: Genome Res. 2020 Nov;30(11):1667–79. doi: 10.1101/gr.260828.120 (PMC7605250; doi:10.1101/gr.260828.120)
Supplement: Supplemental Material [file supp_gr.260828.120_Supplemental_Code_S1.zip › PEPPAN-1.0.5/docs/build/html/index.html]

Welcome to PEPPA’s documentation! — PEPPA 1.0 documentation

# Welcome to PEPPA’s documentation!¶

Contents:

- installation
- quickstart
- parameters
- inputs
- outputs
  - inference=ortholog\_group:<source\_genome>:<exemplar\_gene>:<allele\_ID>:<start & end coordinates of alignment in the exemplar gene>:<start & end coordinates of alignmenet in the genome>

## About PEPPA¶

PEPPA (Phylogeny Enhanced Pipeline for PAn-genome) is a pipeline that can construct a pan-genome from thousands of genetically diversified bacterial genomes.
PEPPA implements a combination of tree- and synteny-based approaches to identify and exclude paralogous genes,
as well as similarity-based gene predictions that support consistent annotations of genes and pseudogenes in individual genomes.

## Citation¶

If you use GrapeTree please cite the pre-print in BioRxiv:

Z Zhou, M Achtman (2020) “Accurate reconstruction of the pan- and core- genomes of bacteria with PEPPA”
bioRxiv, doi: [https://doi.org/10.1101/2020.01.03.894154](https://doi.org/10.1101/2020.01.03.894154)

# Indices and tables¶

- Index
- Module Index
- Search Page

# PEPPA

### Navigation

Contents:

- installation
- quickstart
- parameters
- inputs
- outputs

### Related Topics

- Documentation overview
  - Next: installation

### Quick search

©2020, Zhemin Zhou.
|
Powered by Sphinx 3.0.2
& Alabaster 0.7.12
|
Page source
