# Supplementary material for: Accurate reconstruction of bacterial pan- and core genomes with PEPPAN
Source: Genome Res. 2020 Nov;30(11):1667–79. doi: 10.1101/gr.260828.120 (PMC7605250; doi:10.1101/gr.260828.120)
Supplement: Supplemental Material [file supp_gr.260828.120_Supplemental_Code_S1.zip › PEPPAN-1.0.5/docs/build/html/usage/installation.html]

installation — PEPPA 1.0 documentation

# installation¶

# PEPPA

### Navigation

Contents:

- installation
- quickstart
- parameters
- inputs
- outputs

### Related Topics

- Documentation overview
  - Previous: Welcome to PEPPA’s documentation!
  - Next: Quick Start

### Quick search

©2020, Zhemin Zhou.
|
Powered by Sphinx 3.0.2
& Alabaster 0.7.12
|
Page source
