# Supplementary material for: Accurate reconstruction of bacterial pan- and core genomes with PEPPAN
Source: Genome Res. 2020 Nov;30(11):1667–79. doi: 10.1101/gr.260828.120 (PMC7605250; doi:10.1101/gr.260828.120)
Supplement: Supplemental Material [file supp_gr.260828.120_Supplemental_Code_S1.zip › PEPPAN-1.0.5/docs/build/latex/peppa.pdf]

PEPPA

Release 1.0

Zhemin Zhou

Apr 22, 2020

CONTENTS:

1 installation **1**

2 Quick Start **3**

3 Parameters **5**

4 inputs **9**

5 Outputs **11**

5.1 inference=ortholog_group:<source_genome>:<exemplar_gene>:<allele_ID>:<start & end coordinates of alignment in the exemplar gene>:<start & end coordinates of alignmenet in the genome> . . 11

6 About PEPPA **13**

7 Citation **15**

8 Indices and tables **17**

CHAPTER

ONE

INSTALLATION

CHAPTER TWO

QUICK START

Quick Start (included in example.bash) ``` $ cat example.bash # generate pan-genome prediction using PEPPA python PEPPA.py -P examples/GCF_000010485.combined.gff.gz –min_cds 60 –incompleteCDS s -p examples/ST131 examples/*.gff.gz

generate summaries for PEPPA predicted CDSs and pseudogenes python PEPPA_parser.py -g examples/ST131.PEPPA.gff -s examples/PEPPA_out -m -t -c -a 95

generate summaries for PEPPA predicted CDSs only python PEPPA_parser.py -g examples/ST131.PEPPA.gff -s examples/PEPPA_out -m -t -c -a 95 -P ```

CHAPTER

THREE

PARAMETERS

Usage for PEPPA.py ``` $ python PEPPA.py -h usage: PEPPA.py [-h] [-p PREFIX] [-g GENES] [-P PRIORITY] [-t N_THREAD]

> [-o ORTHOLOGY] [-n] [–min_cds MIN_CDS] [–incompleteCDS INCOMPLETECDS] [–gtable GTABLE] [–clust_identity CLUST_IDENTITY] [–clust_match_prop CLUST_MATCH_PROP] [–nucl] [–match_identity MATCH_IDENTITY] [–match_prop MATCH_PROP] [–match_len MATCH_LEN] [–match_prop1 MATCH_PROP1] [–match_len1 MATCH_LEN1] [–match_prop2 MATCH_PROP2] [–match_len2 MATCH_LEN2] [–match_frag_prop MATCH_FRAG_PROP] [–match_frag_len MATCH_FRAG_LEN] [–link_gap LINK_GAP] [–link_diff LINK_DIFF] [–allowed_sigma ALLOWED_SIGMA] [–pseudogene PSEUDOGENE] [–untrusted UNTRUSTED] [–metagenome] [N [N . . .]]

PEPPA.py (1) Retieves genes and genomic sequences from GFF files and FASTA files. (2) Groups genes into clusters using mmseq. (3) Maps gene clusters back to genomes. (4) Discard paralogous alignments. (5) Discard orthologous clusters if they had regions which overlapped with the regions within other sets that had greater scores. (6) Re-annotate genomes using the remained of orthologs.

positional arguments:

N [REQUIRED] GFF files containing both annotations and sequences. If you have sequences and GFF annotations in separate files, they can also be put in as: <GFF>,<fasta>

optional arguments:

-h, --help show this help message and exit

-p PREFIX, --prefix PREFIX [Default: PEPPA] prefix for the outputs.

-g GENES, --genes GENES [optional] Comma delimited filenames that contain fasta of additional genes.

-P PRIORITY, --priority PRIORITY [optional] Comma delimited, ordered list of GFFs or gene fasta files that are more reliable than others. Genes contained in these files are preferred in all stages.

-t N_THREAD, --n_thread N_THREAD [Default: 20] Number of threads to use. Default: 20

-o ORTHOLOGY, --orthology ORTHOLOGY [Default: nj] Method to define orthologous groups. nj [default], ml (for small dataset) or sbh (extremely large datasets)

-n, --noNeighborCheck [Default: False] Flag to disable checking of neighborhood for paralog splitting.

--min_cds MIN_CDS [Default: 150] Minimum length for a gene to be used in similarity searches.

--incompleteCDS INCOMPLETECDS [Default: ''] Allowed types of imperfection for reference genes. 's': allows unrecognized start codon. 'e': allows unrecognized stop codon. 'i': allows stop codons in the coding region. 'f': allows frameshift in the coding region. Multiple keywords can be used together. e.g., use 'sife' to allow random sequences.

--gtable GTABLE [Default: 11] Translate table to Use. Only support 11 and 4 (for Mycoplasma)

--clust_identity CLUST_IDENTITY minimum identities of mmseqs clusters. Default: 0.9

--clust_match_prop CLUST_MATCH_PROP minimum matches in mmseqs clusters. Default: 0.9

--nucl disable Diamond search. Fast but less sensitive when nucleotide identities < 0.9

--match_identity MATCH_IDENTITY minimum identities in BLAST search. Default: 0.5

--match_prop MATCH_PROP minimum match proportion for normal genes in BLAST search. Default: 0.6

--match_len MATCH_LEN minimum match length for normal genes in BLAST search. Default: 250

--match_prop1 MATCH_PROP1 minimum match proportion for short genes in BLAST search. Default: 0.8

--match_len1 MATCH_LEN1 minimum match length for short genes in BLAST search. Default: 100

--match_prop2 MATCH_PROP2 minimum match proportion for long genes in BLAST search. Default: 0.4

--match_len2 MATCH_LEN2 minimum match length for long genes in BLAST search. Default: 400

--match_frag_prop MATCH_FRAG_PROP Min proportion of each fragment for fragmented matches. Default: 0.3

--match_frag_len MATCH_FRAG_LEN Min length of each fragment for fragmented matches. Default: 50

--link_gap LINK_GAP Consider two fragmented matches within N bases as a linked block. Default: 300

--link_diff LINK_DIFF Form a linked block when the covered regions in the reference gene and the queried genome differed by no more than this value. Default: 1.2

--allowed_sigma ALLOWED_SIGMA Allowed number of sigma for paralogous splitting. The larger, the more variations are kept as inparalogs. Default: 3.

--pseudogene PSEUDOGENE A match is reported as pseudogene if its coding region is less than this amount of the reference gene. Default: 0.8

--untrusted UNTRUSTED FORMAT: l,p; A gene is not reported if it is shorter than l and present in less than p of prior annotations. Default: 300,0.3

--metagenome Set to metagenome mode. equals to "–nucl –incompleteCDS sife –clust_identity 0.99 –clust_match_prop 0.8 –match_identity 0.98 –orthology sbh"

```
```

Usage for PEPPA_parser.py ``` $ python PEPPA_parser.py -h usage: PEPPA_parser.py [-h] -g GFF [-p PREFIX] [-s SPLIT] [-P] [-m] [-t]

[-a CGAV] [-c]

PEPPA_parser.py (1) reads xxx.PEPPA.gff file (2) split it into individual GFF files (3) draw a present/absent matrix (4) create a tree based on gene presence (5) draw rarefraction curves of all genes and only intact CDSs

optional arguments:

-h, --help show this help message and exit

-g GFF, --gff GFF [REQUIRED] generated PEPPA.gff file from PEPPA.py.

-p PREFIX, --prefix PREFIX [Default: Same prefix as GFF input] Prefix for all outputs.

-s SPLIT, --split SPLIT [optional] A folder for splitted GFF files.

-P, --pseudogene [Default: Use Pseudogene] Flag to ignore pseudogenes in all analyses.

-m, --matrix [Default: False] Flag to generate the gene present/absent matrix

-t, --tree [Default: False] Flag to generate the gene present/absent tree

-a CGAV, --cgav CGAV [Default: -1] Set to an integer between 0 and 100 to apply a Core Gene Allelic Variation tree. The value describes % of presence for a gene to be included in the analysis. This is similar to cgMLST tree but without an universal scheme.

-c, --curve [Default: False] Flag to generate a rarefraction curve.

```
```

CHAPTER

FOUR

INPUTS

CHAPTER

FIVE

OUTPUTS

Outputs for PEPPA.py There are two final outputs for PEPPA.py:

1. <prefix>.PEPPA.gff

   This file includes all pan-genes predicted by PEPPA in GFF3 format. Intact CDSs are assigned as "CDS", disrupted genes (potential pseudogenes) are assigned as "pseudogene" and suspicious annotations that are removed are described as "misc_feature" entries.

- If any of the predicted CDSs and psueogenes overlaps with old gene predictions in the original GFF files, the old gene is described in an attribute named "old_locus_tag" of the entry.
- Each gene and pseudogene is assigned into one of the orthologous groups. This orthologous group is described in "inference" field in a format of:

5.1 inference=ortholog_group:<source_genome>:<exemplar_gene>:<allele_ID> & end coordinates of alignment in the exemplar gene>:<start & end coordinates of alignmenet in the genome>

2. <prefix>.alleles.fna

   This file contains all the unique alleles of all pan genes predicted by PEPPA.

Outputs for PEPPA_parse.py PEPPA_parse.py generates:

1. <prefix>.gene_content.matrix or <prefix>.CDS_content.matrix

A matrix of gene presence/absence in all genomes.

2. <prefix>.gene_content.nwk or <prefix>.CDS_content.nwk

A tree built based on gene presence/absence in all genomes.

3. <prefix>.gene_content.curve or <prefix>.CDS_content.curve

The rare-fraction curves for the pan-genome and core-genome

4. <prefix>.gene_CGAV.tree or <prefix>.CDS_CGAV.tree

Core Genome Allelic Variation trees based on the sequence differences of the core genes. This is similar to but should not be treated as a cgMLST scheme, because the genes included in the analysis depend on the genomes. The result of CGAV analysis is not comparable across different analyses.

CHAPTER

SIX

ABOUT PEPPA

PEPPA (Phylogeny Enhanced Pipeline for PAn-genome) is a pipeline that can construct a pan-genome from thousands of genetically diversified bacterial genomes. PEPPA implements a combination of tree- and synteny-based approaches to identify and exclude paralogous genes, as well as similarity-based gene predictions that support consistent annotations of genes and pseudogenes in individual genomes.

CHAPTER

SEVEN

CITATION

If you use GrapeTree please cite the pre-print in BioRxiv:

Z Zhou, M Achtman (2020) "Accurate reconstruction of the pan- and core- genomes of bacteria with PEPPA" bioRxiv, doi: [https://doi.org/10.1101/2020.01.03.894154{]}(https://doi.org/10.1101/2020.01.03.894154)

CHAPTER EIGHT

INDICES AND TABLES

- genindex
- modindex
- search
